# Does Retirement Age Impact Functional Limitations in Later Life?

**DOI:** 10.1093/geroni/igab046.3684

**Published:** 2021-12-17

**Authors:** Ziyao Xu

**Affiliations:** Miami University, Oxford, Ohio, United States

## Abstract

The US government is gradually shifting the full retirement age in Social Security to age 67. However, previous studies suggest that this shift could negatively impact the mental and physical health of retirees. To understand the potential impact of raising the full retirement age on the functional health of retirees, this longitudinal study examined changes in physical functioning over time in retirees by age at retirement. Twelve waves of the Health and Retirement Study (1994 – 2018) were used. A total of 8,261 retirees was included. The retirement age was a categorical variable: very early age (<62), early age (62-64), traditional age (65-67), and late age (>67). Physical functioning was measured using 15 Activities of Daily Living and Instrumental Activities of Daily Living. A GEE model was used to assess the relationship between the retirement age category and the number of functional limitations. In the adjusted model, after controlling for all the other variables including baseline health and functioning, late retirement was associated with an 8.9% increased risk of functional limitations compared to traditional age retirement (IRR: 0.91, 95% CI:0.84 –0.98). Compared to late retirees, the risk of functional limitations was increased by 28.6% in very early age retirees (IRR: 1.29, 95% CI:1.21–1.36). Compared to those retiring at traditional retirement age, those retiring late, after 67, have increased the risks of functional limitations. Although levels of disability could influence age of retirement, these results suggest that for some workers efforts to increase age of full retirement, could have negative effects.

